# The Effect of Water Hardness and pH on the Efficacy of Peracetic Acid and Sodium Hypochlorite against SARS-CoV-2 on Food-Contact Surfaces

**DOI:** 10.3390/foods12162981

**Published:** 2023-08-08

**Authors:** Julianna N. Morris, Malak A. Esseili

**Affiliations:** Center for Food Safety, Department of Food Science and Technology, University of Georgia, Griffin, GA 30223, USA; julianna.morris@uga.edu

**Keywords:** SARS-CoV-2, peracetic acid, sodium hypochlorite, water hardness, pH, food-contact surfaces, wet markets, wildlife hunting

## Abstract

Sodium hypochlorite (NaOCl) and peracetic acid (PAA) are commonly used disinfectants with a maximum recommended concentration of 200 ppm for food-contact surfaces. The objectives of this study were to assess the effect of pH and water hardness on NaOCl and PAA efficacy against SARS-CoV-2 on stainless steel (SS). The two disinfectants were prepared at 200 ppm in water of hardness 150 or 300 ppm with the final pH adjusted to 5, 6, 7, or 8. Disinfectants were applied to virus-contaminated SS for one minute at room temperature following the ASTM E2197 standard assay. SARS-CoV-2 infectivity was quantified using TCID50 assay on Vero-E6 cells. In general, increasingly hard water decreased the efficacy of NaOCl while increasing the efficacy of PAA. Hard water at 300 ppm significantly increased virus log reduction with PAA at pH 8 by ~1.5 log. The maximum virus log reductions were observed at pH 5 for both NaOCl (~1.2 log) and PAA (~2 log) at 150 and 300 ppm hard water, respectively. In conclusion, PAA performed significantly better than NaOCl with harder water. However, both disinfectants at 200 ppm and one minute were not effective (≤3 log) against SARS-CoV-2 on contaminated food-contact surfaces, which may facilitate the role of these surfaces in virus transmission.

## 1. Introduction

In December 2019, an outbreak of severe pneumonia was reported by China to the World Health Organization (WHO). Of the first 41 patients hospitalized, 66% had direct exposure to wet markets, specifically the Huanan wholesale seafood market in Wuhan, China [1]. The causative agent was later identified to be the severe acute respiratory syndrome-coronavirus-2 (SARS-CoV-2), and the disease was called COVID-19. Food animals sold alive or slaughtered for fresh meat are a common practice in these wet markets [2]. For example, between 2017 and 2019, more than 47,000 animals belonging to 38 species, including illegally caught wildlife, were sold in wet markets in Wuhan, China [3]. These animals are usually kept in cages under poor hygienic conditions, including the presence of animal feces in the vicinity [2,3]. Many of the animals sold at these markets were later shown to be susceptible to SARS-CoV-2 infection [1]. Also, a wide range of environmental and food-contact surfaces collected from Huanan Market tested positive (33 of 585) for SARS-CoV-2 by RT-qPCR [1]. For example, a wagon surface, scale, trash cart, door surface, freezer surface, fish packing surface, hair/feather removal machine, metal cage, gloves, foam dispenser, shoe bottoms, wall surfaces as well as dirty water and blood from ground surfaces were found positive for SARS-CoV-2 [1]. The latter indicated that proper disinfection of environmental and food-contact surfaces was limited at these markets, which may facilitate the transmission of zoonotic disease to humans in these settings [2]. The Huanan wet market is now recognized as the epicenter of the COVID-19 pandemic [1].

In Western countries, central slaughtering of food animals is adopted which limits direct exposure of the population to food animals and, consequently, to zoonotic pathogens. Nevertheless, early during the pandemic, many meat processing facilities across the United States experienced large outbreaks of SARS-CoV-2. Almost 3% of the workforce from over 100 processing facilities became infected with SARS-CoV-2; resulting in meat shortages, economic loss, and the death of 20 individuals [4,5]. While these hotspots for virus transmission were eventually controlled, another less apparent mode of SARS-CoV-2 transmission from wildlife animals such as white-tailed deer was gaining recognition [6,7]. Recent studies showed that SARS-CoV-2 with multiple variants of concern was prevalent in wild deer at about 30–40% prevalence [8,9] and high infectious viral load can be present in multiple deer tissues [7]. This could potentially lead to spill back to humans, knowing, for example, that there is huge population of deer (>30 million) and deer hunters (>10 million) in the US generating billions of dollars annually for the US economy [10]. Human-to-deer and deer-to-deer transmission of SARS-CoV-2 are now recognized; however, deer to human is less investigated and can theoretically happen from direct exposure to infected deer or through processing of hunted deer and the associated contamination of food-contact surfaces. Furthermore, unhygienic food-contact surfaces are recognized as vehicles of infectious disease transmission [11], and laboratory studies have shown that SARS-CoV-2 artificially inoculated on meat or food-contact surfaces can persist for prolonged periods of time depending on the temperature [12,13,14]. Multiple factors can affect the survival of SARS-CoV-2 on surfaces such as virus load, the presence of organic matrix, temperature, and relative humidity [15]. For example, at room temperature (~25 °C) and relative humidity of 35–50%, SARS-CoV-2 in a tripartite soil load deposited on SS showed 1-log reduction after 96–143 h [16]. In another study, transfer of SARS-CoV-2 from SS to fingers was found to be high, ranging from 19.5–100% depending on the prevailing relative humidity [17]. Another study showed that the transfer of SARS-CoV-2 from surfaces to fingers was substantial when virus droplets were wet, while transfer occurred at a lower rate after droplets evaporated [18]. Hence, implementing proper hygiene measures is important to safeguard against the possibility of food-contact surfaces spreading SARS-CoV-2.

Sodium hypochlorite (NaOCl) is the most commonly used disinfectant by the food industry [19,20]. NaOCl is a strong oxidizing agent with broad antimicrobial properties and has been in use for over 100 years. However, peracetic acid (PAA) has a stronger oxidation potential than NaOCl [21] and is emerging as an alternative to chlorination due to its broad antimicrobial activity and low-toxicity byproducts [22]. In the food industry, PAA is mainly used in food processing and handling to disinfect food-contact surfaces and recirculated flume water and as a sanitizer for fruits, vegetables, meat, and eggs [21]. Certain factors can either optimize or reduce the efficacy of disinfectants, including pH, temperature, organic matter, water hardness, disinfectant concentration (in-use dilution), contact time with the pathogen, and surface type [19,20]. It is well established that the pH of NaOCl affects its antimicrobial action [19]. When diluted in water, NaOCl dissociates into two chemical species: hypochlorous acid (HOCl) and hypochlorite ion (OCl^−^). The HOCl has greater antimicrobial action than OCl^−^ and is the dominant species at pH < 7; with the highest proportion at pH 4–5. OCl^−^ dominates at pH > 7, and toxic chloride gas starts to accumulate at pH < 4 [20]. In comparison, PAA occurs as an aqueous mixture under equilibrium with acetic acid, hydrogen peroxide, and water [23] and is thought to be more germicidal at acidic pH [20,24]. NaOCl’s mechanisms of action are thought to be through the HOCl molecule which disrupts peptide bonds and thiol groups on proteins through oxidation and chlorine exchange [25]. The HOCl molecule can penetrate negatively charged membranes and causes an exchange of hydrogen with chlorine. This exchange causes the membrane to break and thus microorganisms become inactivated [26]. PAA is theorized to denature proteins, oxidize sulfhydryl and sulfur bonds, and permeabilize cell walls or viral envelopes [27]. Furthermore, it is thought that increases in temperature, up to 52 °C, increase NaOCl efficacy [19]. The latter is attributed to factors not related to hypochlorous acid proportion but to an increase in the pH of the solution and lowering of surface tension [19]. For PAA, it is reported that it can be used at a wide temperature range (0–40 °C); however, some reports mentioned higher temperatures (20–25 vs. 4–5 °C or 43–46 vs. 22 °C) increased the efficacy of PAA [24,28], while others reported a decrease in the antimicrobial efficacy at 35 °C and no difference between 15 and 25 °C if the solution was at pH 8 or 9 [29]. For organic matter, it is widely accepted that the efficacy of NaOCl decreases with increases in organic matter content, because this generates organic chloramines which have little antimicrobial efficacy [20]. Meanwhile, PAA is considered less reactive to organic matter and as such its efficacy is, comparatively, partially affected by organic matter depending on the concentration used [30,31]. Water hardness can directly affect the efficacy of disinfectants, because calcium and magnesium can combine with bicarbonates to form difficult to remove films especially under heat and alkaline pH [31]. The way a combination of factors can enhance or reduce the disinfectant efficacy is not well known, especially for NaOCl and PAA against SARS-CoV-2 on surfaces. This may lead to failure in disinfection of food-contact surfaces and spread of SARS-CoV-2 [11].

The objective of this study was to assess the effect of pH and hard water on the efficacy of NaOCl and PAA against SARS-CoV-2 on food-contact surfaces. Toward this objective, we used NaOCl and PAA at 200 ppm, the maximum FDA-recommended concentration on food-contact surfaces, without an additional rinse step [21]. Stainless steel (SS) is by far the most preferred food-contact surface, because it is a corrosion-resistant metal and easier to clean, hence it is specified by many regulatory designs and construction standards [32]. We used SS as specified in the ATM standard E2197 assay for testing disinfectant on surfaces and their standard composition of organic matter (mucin, bovine serum albumin, and tryptone) [33]. The desired contact time for a disinfectant on food-contact surfaces is 30 s to show a 5-log reduction in bacteria [31]. However, the contact time is not well defined for viruses, especially for SARS-CoV-2, but a 3-log reduction is required by EPA for a disinfectant to be labeled effective against SARS-CoV-2 on inanimate surfaces [34]. To comparatively assess the effect of pH and water hardness on PAA and NaOCl efficacies, we used a fixed contact time of 1 min. According to the USGS, hard water contains 121–180 ppm of CaCO_3_ and very hard water contains >180 ppm CaCO_3_ [35]. Subsequently, we tested two levels of hard water, 300 ppm which is recommended by ATSM E2197 and 150 ppm which is typical of what is found in tap water [28]. A range of pH 5–8 was tested to include the natural pH of PAA (pH ~5) and that of chlorine (pH ~8) in hard water at room temperature (~22–25 °C).

## 2. Materials and Methods

### 2.1. Virus Propagation and Cell Culture

The USA reference strain SARS-CoV-2 USA-WA 1/2020 (BEI resources NR-52281) was propagated in African green monkey kidney cells (Vero-E6 ATCC CRL-1586) as described previously [36]. Handling of SARS-CoV-2 was carried out under strict BSL3 biosafety protocols at the Center for Food Safety BSL3 laboratory. Briefly, one- or two-day-old 90% confluent cells were used to prepare virus stocks using a multiplicity of infection of 0.01. Infection media consisted of DMEM supplemented with 2% heat-inactivated fetal bovine serum and 1% antibiotic–antimycotic cocktail. Harvesting the virus was carried out at 72 h post-infection. Infected cells were collected from the flasks and centrifuged at 450× *g* for 5 min at 4 °C to pellet the cell debris, while supernatants containing the virus were ultrafiltered through Amicon 100 KDa Ultra-15 centrifugal devices (MilliporeSigma, St. Louis, MO, USA) immediately after harvest to concentrate the virus 10 times, while semi-purifying it from cell culture lysates [37]. The latter would also overcome the buffering effect of infection media in virus stocks on NaOCl. An aliquot of the virus was immediately titrated as described below. The original viral titer generated was ~7 log_10_ TCID_50_/mL, while the ultrafiltered virus titer was ~8 log_10_ TCID_50_/mL.

### 2.2. Preparation of Disinfectant Solutions

Sodium hypochlorite solutions were made by diluting commercial bleach (7.5% NaOCl, The Chlorox Company, Oakland, CA, USA) in sterile water using fresh (unopened) commercial bleach for every experiment. The free available chlorine was measured in all NaOCl solutions using Hydrion Chlorine Test Paper (Micro essential laboratory, New York, NY, USA). Peracetic acid solutions were made by diluting concentrated peracetic acid (17% PAA with 26% H_2_O_2_, Peroxychem LLC, Philadelphia, PA, USA) in sterile water.

Disinfectants at 200 ppm were prepared in 300 ppm hard water. The AOAC guideline for making hard water, measured in mg/L of CaCO_3_, was followed [38]. Two solutions were used to make the hard water. The first solution was composed of 0.98 M anhydrous MgCl_2_ and CaCl_2_ (Sigma-Aldrich, Burlington, MA, USA) dissolved in boiled deionized water. The second was composed of 0.66 M NaHCO_3_ (Sigma). Both solutions were sterilized by membrane filtration using a 0.22 μm nitrocellulose filter (Millipore). Per 100 ppm of hard water, 1 mL of solution one and 4 mL of solution two were added to 995 mL of sterile water. Disinfectants were then adjusted to pH from 5–8 after dilution of disinfectant using 1M HCl or 1M NaOH and measured using Orion Star A211 (Thermo Scientific, Waltham, MA, USA). PAA solutions in hard water had a natural pH ~5 and those of NaOCl had pH ~8. Hard water and disinfectant solutions were made fresh for every experiment.

### 2.3. Quantitative Disk Carrier Test Method ASTM E2197

The surfaces used were stainless steel disks of type 304, no. 4 finish (Washington Specialty Metal, Athens, GA, USA). Stainless steel disks were placed inside wells of 6-well plates and inoculated with the virus. The ultrafiltered viruses were directly diluted in soil load and 10 μL of the resulting virus in soil suspension was inoculated on the disks. Soil load with virus in volumes of 500 μL was composed of a tripartite mixture of 35 μL tryptone (Sigma-Aldrich, St. Louis, MO, USA), 25 μL bovine serum albumin (BSA) (HyClone, Logan, UT, USA), 100 μL mucin from bovine submaxillary glands (Sigma-Aldrich St. Louis, MO, USA), and finally 340 μL virus. Tryptone and BSA solutions were prepared by dissolving 0.5 g each in separate 10 mL amounts of phosphate buffer solution (PBS). Meanwhile, the mucin solution was prepared by dissolving 0.04 g in 10 mL PBS. All three solutions were sterilized by membrane filtration using 0.22 μm nitrocellulose filters (VWR, Radnor, PA, USA).

The virus-contaminated disks were allowed to dry at room temperature for 45 min inside a biosafety cabinet. Fifty microliters of disinfectants was added to virus-inoculated disks. The contact time was one minute. Disinfectants were neutralized with 50 µL of 0.025 M sodium thiosulfate (Sigma-Aldrich) for NaOCl [39] or Dey/Engley neutralizing broth (Thomas Scientific, Swedesboro, NJ, USA) for PAA [40]. Disks were then immediately transferred to 50 mL Falcon tubes containing 900 μL of cell culture infection media and vortexed at medium speed for 1 min. Eluates were transferred to 1.5 mL tubes and tested immediately using the TCID50 assay as described below. Controls were included for verification of neutralization (disinfectant and neutralizer were combined, held for one minute, then the virus was added) and the assessment of the effect of neutralizer on virus (only virus and neutralizers combined). Cytotoxicity controls were included for the disinfectants, neutralizer, and disinfectant and neutralizer combined. A negative control (without virus added) and positive control (virus only) were included in each experiment. All experiments were performed at room temperature. After using these disks, they were sterilized by spraying with 10% Micro-Chem Plus (National Chemical Laboratories, Philadelphia, PA, USA) and autoclaved at 121 °C for 120 min to inactivate SARS-CoV-2. After autoclaving, disks were sprayed with 70% ethanol and rinsed twice with deionized sterile water to remove Micro-Chem residue before being autoclaved a second time and re-used.

### 2.4. Quantification of Virus Infectivity Using TCID_50_ Assay

Quantification of virus titers was performed using the 50% tissue culture infectious dose (TCID50) assay. Briefly, ten-fold serially diluted samples in infection media were added to four wells of 1–2-day-old Vero-E6 cells in a 96-well tissue culture plate. After 4–5 days of incubation, the cells were observed for cytopathic effect (CPE). The titer was determined from the dilutions where 50% of the wells showed CPE and was then calculated based on the Reed and Muench method [41]. Negative and positive controls were included in every experiment as described above. Neither the neutralizers nor the disinfectant combined with neutralizers had any significant effect on SARS-CoV-2 titers. However, PAA and NaOCl had a limited and infrequent cytotoxic effect on Vero-E6 cells at the undiluted concentrations. Therefore, the detection limit in the TCID50 assay was set at 1.8 log_10_ TCID_50_/mL when cytotoxicity was observed.

### 2.5. Statistical Analyses

All experiments were performed independently at least two times; with at least three to four technical replicates. The entire dataset was transformed to log_10_. Means and standard errors were calculated from all replicates. The log_10_ reductions were calculated by subtracting virus titers of all technical replicates for a specific treatment from the average virus titer of the control (no treatment). GraphPad Prism version 5 (GraphPad Software, San Diego, CA, USA) was used for all statistical analyses. Data were expressed as mean ± standard error. Two-way analysis of variance (ANOVA) followed by a Bonferroni post-test was used to compare means. Differences in means were considered significant when the *p* value was less than 0.05 and are denoted in the figures and tables by either different letters, asterisks, or bolded numbers.

## 3. Results

### 3.1. Effect of Hard Water and pH of 200 ppm NaOCl against SARS-CoV-2 on Stainless Steel at One Minute Contact Time

**Figure 1:**
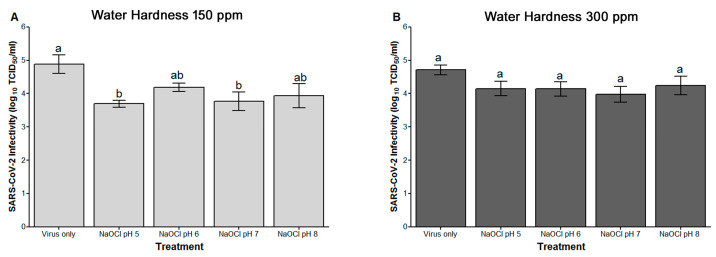
Effect of hard water and pH of the final NaOCl disinfectant solution on SARS-CoV-2 infectivity (log_10_ TCID_50_/mL). The disinfectant was prepared in (**A**) 150 ppm or (**B**) 300 ppm hard water and the pH of the final solution was adjusted to 5, 6, 7, or 8. SARS-CoV-2 was prepared in tripartite soil load and inoculated on SS disks according to ASTM E2197 standard method. Disinfectant was applied to SS disks for a 1 min contact time. Data are reported as means ± standard error. Means with different letters differ significantly.

SARS-CoV-2 on SS disks treated with NaOCl (200 ppm) prepared in water of hardness of 150 ppm and adjusted to pH 5 and 7 showed significantly lower infectivity titers as compared to virus on SS treated with water (Figure 1A). In contrast, when NaOCl (200 ppm) was prepared in water of hardness of 300 ppm, none of the NaOCl treatments at any pH tested showed any significant difference in comparison to virus on SS treated with water (Figure 1B). The maximum log reduction was achieved with NaOCl at pH 5 using water of 150 ppm hardness (1.18 ± 0.1 log) (Table 1). However, two-way ANOVA revealed that the virus log reductions at any pH tested were not significantly different from each other’s (Table 1). In general, increasing the water hardness for NaOCl resulted in lower log reductions across all pH levels tested (Table 1). Two-way ANOVA revealed that pH and hard water explain 2.8 and 6% of the total variance in the log reduction data and that this effect is significant for water hardness only. Overall, when using very hard water (300 ppm) and not adjusting the pH of NaOCl (~pH 8), a ≤ 0.5-log reduction in SARS-CoV-2 on SS occurred in one minute, whereas, when adjusting the pH to 5 and using water with less hardness (150 ppm), a 1-log reduction (90%) was achieved. 

### 3.2. Effect of Hard Water and pH of 200 ppm PAA against SARS-CoV-2 on Stainless Steel at One Minute Contact Time

**Figure 2:**
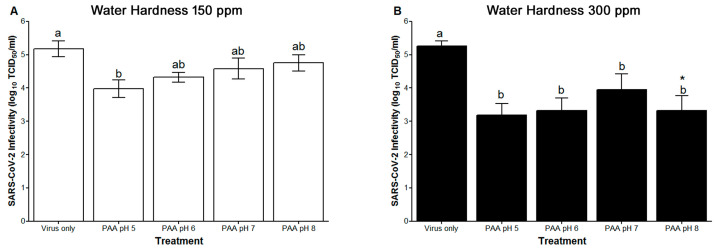
Effect of hard water and pH of the final PAA disinfectant solution on SARS-CoV-2 infectivity (log_10_ TCID_50_/mL). The disinfectant was prepared in (**A**) 150 ppm or (**B**) 300 ppm hard water and the pH of the final solution was adjusted to 5, 6, 7, or 8. SARS-CoV-2 was prepared in tripartite soil load and inoculated on SS disks according to ASTM E2197 standard method. Disinfectant was applied to SS disks for a 1 min contact time. Data are reported as means ± standard error. Means with different letters differ significantly. The asterisk (*) sign indicates significant difference between the two hardness levels of a specific pH treatment.

When SS disks contaminated with SARS-CoV-2 were treated with a 200 ppm PAA solution prepared in 150 ppm hard water, only PAA at a final pH of 5 showed a significant effect on SARS-CoV-2 infectivity titer in comparison to virus disks treated with water (Figure 2A). This effect gradually diminished as the pH of PAA solution was increased to 8 (Figure 2A). In contrast, SARS-CoV-2-contaminated disks treated with PAA solutions prepared in 300 ppm hard water and adjusted to a final pH of 5, 6, 7, and 8 showed significant effects on SARS-CoV-2 on SS (Figure 2B). The maximum log reduction obtained with the 200 ppm PAA solution prepared in 150 ppm hard water was at pH 5 (1.19 ± 0.26 log) (Table 1). The maximum log reduction observed with the PAA solution prepared in 300 ppm hard water was again obtained at pH 5 (2.08 ± 0.3 log) (Table 1). However, in both cases, these log reductions were not significantly different from log reductions obtained with other tested pHs (Table 1). Two-way ANOVA revealed that pH and hard water explain 5.5 and 26.8% of the total variance in the log reduction data and that this effect was significant for water hardness only. In general, increasing the water hardness for PAA resulted in higher log reductions across all pH levels tested (Table 1). This effect was significant at pH 8 where 300 ppm showed an additional ~1.5 increase in virus reduction as compared to 150 ppm water hardness (Table 1). Overall, not adjusting the pH of PAA (~pH 5) and using very hard water (300 ppm) would result in ~2-log reduction (99%) in SARS-CoV-2 on SS in one minute.

### 3.3. Comparing NaOCl to PAA Effect on SARS-CoV-2 on Contaminated Stainless Steel

In the NaOCl experiments, the infectivity titer of the viruses from disks receiving water of 150 ppm hardness (4.88 ± 0.28 log) was not significantly different from those receiving 300 ppm hard water (4.71 ± 0.14 log) (Figure 1A,B). Similarly, in the PAA experiments, SARS-CoV-2 on SS treated with 150 ppm hard water was not significantly different from that treated with 300 ppm hard water (5.17 ± 0.23 and 5.26 ± 0.15 log, respectively) (Figure 2A,B). In addition, these results were not significantly different from those obtained from the NaOCl experiments. When comparing virus log reduction between PAA to NaOCl, no significant differences were observed when these disinfectants were prepared in water of 150 ppm hardness, regardless of the final pH (Table 1). In contrast, when 300 ppm hard water was used, a significantly higher log reduction was observed for PAA in comparison to NaOCl under all pH levels except at pH 7 (Table 1). Overall, two-way ANOVA revealed a significant effect for the type of disinfectant at 300 ppm water hardness but not at 150 ppm water hardness.

## 4. Discussion

In this study, we investigated two factors, namely pH and water hardness that can affect the efficacy of NaOCl and PAA against SARS-CoV-2 on food-contact surfaces. A fixed contact time (one minute), temperature (~25 °C), organic matter (ASTM tripartite composition), and disinfectant concentration (200 ppm as per FDA guidelines for food-contact surfaces) were used. First, it was established that SARS-CoV-2 infectivity was not significantly affected by the range of pH of 5–8 used in our study and a one-minute contact time, as neither a twenty-minute [42] nor one-hour [43] incubation at room temperature had had any significant effects. It would take 24 h of virus incubation at room temperature with buffers of pH 5 to 8 to observe a significant decrease in virus infectivity of ~1 and 1.2 log, respectively [44]. Second, there are no previous studies assessing the effect of hard water by itself on virus infectivity. However, our data revealed no significant difference in SARS-CoV-2 infectivity on SS disks treated with water with hardness of 150 ppm or 300 ppm. Therefore, the reductions in virus infectivity observed in our study within the one-minute contact time were due to the action of the disinfectants, not pH or hard water by themselves. Previous studies investigating the virucidal effect of NaOCl and/or PAA against SARS-CoV-2 were mostly carried out using suspension assays, with high disinfectant concentrations (≥200 ppm up to 5000 ppm) or contact time (≥1 to 30 min), on high-touch surfaces (such as door knobs, toilets, sinks, hospital surfaces, etc.) or using surrogate coronaviruses (reviewed by [45]). Only one study examined NaOCl against SARS-CoV-2 on food-contact surfaces using the maximum concentration recommended by the FDA, 200 ppm [13]. Specifically, on SS, NaOCl at 200 pm showed a ~1.4-log reduction in SARS-CoV-2 infectivity in 1 min using virus in standard tripartite soil composition and NaOCL diluted in 375 ppm hard water. While the previous study did not mention the pH of the final NaOCl solution, our data similarly showed that NaOCl in 300 ppm hard water was not effective (~0.5- to 0.8-log reduction) against SARS-CoV-2 on SS. No previous studies can be found for PAA at 200 ppm against SARS-CoV-2 on food-contact surfaces while investigating factors that can optimize or reduce the efficacy of these commonly used disinfectants.

Studies on the effect of water hardness and pH on the antimicrobial efficacy of NaOCl and PAA are limited and were mostly carried out with bacterial pathogens either on fruits and vegetables or in process waters but not on food-contact surfaces contaminated with viruses. In these studies, it was established that the final pH of NaOCl affects its antimicrobial activity. This is because HOCl is the dominant chlorine species in NaOCl solutions with pH 4–7 [19,20]. HOCl is the strongest oxidizer of the chlorine species, thus being preferred in disinfectant solutions [25,46]. This is in agreement with NaOCl use in the food processing industry, where NaOCl in produce wash water must be adjusted to pH < 6.5 to maximize the active form of chlorine in water [30]. Furthermore, a previous study found that NaOCl at 500 ppm was more effective at pH 5.5–7, with maximum inactivation at pH 7 (5-log reduction) as compared to alkaline pH 8 (~2.5-log reduction) against *Bacillus* spores [46]. While the authors used deionized water typically of low hardness to dilute their disinfectants, we also obtained a higher log reduction for NaOCl at pH 5 and 7 (~1.1 log) when using the lower hard water level. At pH ≥ 7.6 (the pKa of chlorine), a weaker chlorine species begins to predominate, the hypochlorite anion (OCl^−^) [19,20], which may explain our NaOCl data that showed that minimal log reduction occurred at pH 8, especially in 300 ppm water hardness. This, in addition to the interaction of divalent cations with carbonate at alkaline pH, reduces the efficacy of NaOCl at 300 ppm water hardness [31].

Although PAA is thought to be germicidal across pH 0–7.5 [20], the pH value at which PAA shows maximum pathogen reduction may depend on the target application and the pathogen. For example, the adjustment of a PAA solution (80 ppm prepared in tap water of hardness 140 ppm) from pH 6.3 to pH 2.3 and 3.8 did not show any significant effects on its efficacy against *Listeria monocytogenes* on apples; resulting in ~1.7-log reduction in 2 min [28]. In a suspension assay, a 300 ppm PAA solution incubated for 30 min with *Bacillus subtilis* spores at pH 8 and pH 9 showed little inactivation (1- and 0-log reduction) but was more effective at pH 5 (2-log reduction) [46]. In produce wash water, PAA at pH 5 provided the most log reduction in *E. coli* O157: H7 (~5 log) compared to pH 6.5 and 9 [47]. These studies are in agreement with our results, where PAA at pH 5 achieved the highest log reduction value in SARS-CoV-2 infectivity titers under both hard water levels (~1.2 log at 150 ppm and 2 log at 300 ppm).

While there are no studies addressing the effect of water hardness of NaOCl against SARS-CoV-2, a previous study reported that water hardness reduced NaOCl antimicrobial activity. Specifically, an increase in the hardness of water from 50–100 ppm to 200 ppm of an NaOCl solution (~65 ppm) was found to significantly reduce the inactivation of *E. coli* O157:H7 (6.5 to 4.5 log) in a suspension assay at one minute contact time [35]. In general, for NaOCl, we noted a reduced efficacy at all pH levels tested when hard water was raised to 300 ppm. In contrast, we observed that the overall PAA efficacy against SARS-CoV-2 was enhanced by higher hardness. This effect was significant for mean virus log reduction for PAA at pH 8 (300 vs. 150 ppm water hardness). A previous study reported that variation in water hardness (20, 140, and 460 ppm) of a PAA solution (80 ppm) did not affect its efficacy against *Listeria monocytogenes* on apples within 2 min contact time at 22 °C [28]. However, in that study, the pH of PAA in waters of different hardnesses was not mentioned except for the pH at a hardness level of 140 ppm which was 6.3. It is reported that a pH of PAA above the pKa value of 8.2 may reduce its stability and efficacy depending on the concentration [48], which may explain why our lowest observed log reductions for PAA were at pH 8 and 150 ppm hard water. It is also reported that lower hardness levels cause wide fluctuation in the pH of PAA [49]. These fluctuations cause the equilibrium between peracetic acid and hydrogen peroxide to shift [48]. The latter may explain our data that showed that PAA solution in less hard water was less efficacious than in harder water, especially for pH 8. Malchesky et al. [24] mentioned that PAA can have an extended germicidal effect at high concentrations under alkaline pH. This idea is further corroborated by a recent study showing an extended PAA efficacy through pH 8.2 to 10 as compared to unadjusted pH (~4.5–6) when PAA was used at higher concentrations (500 ppm) and longer contact times (60 min) against bacterial pathogens on chicken wings [50]. Although the hard water level was not reported in these studies, it can be a factor leading to this enhanced efficacy. More research is needed to understand the mechanism by which more divalent cations in hard water enhanced PAA efficacy against SARS-CoV-2, specifically at alkaline pH.

Overall, PAA performed similarly to NaOCl at lower water hardness; however, at a higher hard water level, PAA was better than NaOCl, showing up to 2-log reduction in SARS-CoV-2 infectivity across multiple pH values. A previous study also showed that in a direct comparison between NaOCl and PAA, the efficacy of PAA was far better [28]. Specifically, when 100 ppm NaOCl at an adjusted pH of 6.8 was sprayed on apples inoculated with *Listeria monocytogenes* a maximum of 0.9-log reduction was observed in 2 min, while PAA at 80 ppm and final pH of 6.3 showed a 1.7- to 2.7-log reduction [28]. Other than the differential effects of hard water on NaOCl and PAA discussed above, organic matter is expected to decrease the efficacy of NaOCl in comparison to PAA. In a direct comparison between PAA and NaOCl against *Bacillus subtilis* spores, NaOCl (500 ppm) sporicidal activity was completely inhibited in the presence of organic matter (>2% serum), while PAA (300 ppm) required much higher organic load (25% serum) to be completely inhibited [51].

## 5. Conclusions

Taken together, although there were no overall significant differences among NaOCl pH levels of 5 to 8 in terms of virus log reduction, our data suggested that NaOCl, when pH adjusted after dilution in water, would achieve higher values for virus log reduction on SS. However, from a practical standpoint, households using bleach for disinfection of food-contact surfaces do not adjust the pH of the diluted NaOCl, which is around pH 8. This is in accordance with disinfectant label instructions and EPA regulations [19,52]. The latter would result in about 0.5–1log inactivation of SARS-CoV-2 with NaOCl. In contrast, PAA diluted in hard water would result in 1.2–2-log virus reduction without any pH adjustments. The EPA requires a 3-log reduction in virus infectivity for a disinfectant to be considered an effective virucidal on inanimate surfaces [27]. In settings such as wet markets with poor hygiene conditions or wildlife hunting such as deer hunting, current standards of using either NaOCl or PAA at 200 ppm with a quick one-minute contact time are not enough, especially when these surfaces are not pre-cleaned before disinfection. Therefore, targeted preventive measures need to be devised and validated for premises handling, slaughtering, and processing meat from animals susceptible to SARS-CoV-2 infection to prevent further spread of the contamination via food-contact surfaces.

## Figures and Tables

**Table 1:** Effect of water hardness and pH for NaOCl and PAA on the log reduction of SARS-CoV-2 infectivity (TCID_50_/mL) at one minute contact time. The disinfectants were used at 200 ppm on SS disks. Data are reported as means ± standard error. Means with different letters differ significantly within the hardness level of a specific disinfectant. The asterisk (*) sign indicates significant difference between the two hardness levels of a specific pH treatment. Bold numbers indicate significant differences between NaOCl and PAA for specific water hardness levels.

	Virus Reduction (Log_10_TCID_50_/mL)		
Disinfectant	Hardness	150 ppm	300 ppm
NaOCl	pH 5	1.18 ± 0.1 a	0.6 ± 0.2 a
	pH 6	0.7 ± 0.11 a	0.6 ± 0.2 a
	pH 7	1.12 ± 0.27 a	0.79 ± 0.2 a
	pH 8	0.95 ± 0.36 a	0.52 ± 0.26 a
PAA	pH 5	1.19 ± 0.26 a	**2.08 ± 0.34** a
	pH 6	0.85 ± 0.15 a	**1.93 ± 0.37** a
	pH 7	0.63 ± 0.3 a	1.36 ± 0.44 a
	pH 8	0.47 ± 0.22 a	**1.93 ± 0.44** a *

## Data Availability

The datasets generated during and/or analyzed during the current study are available from the corresponding author upon reasonable request.
